# Suicidal Ideation Profiles in Patients with Fibromyalgia Using Transdiagnostic Psychological and Fibromyalgia-Associated Variables

**DOI:** 10.3390/ijerph18010209

**Published:** 2020-12-30

**Authors:** Jorge L. Ordóñez-Carrasco, María Sánchez-Castelló, Elena P. Calandre, Isabel Cuadrado-Guirado, Antonio J. Rojas-Tejada

**Affiliations:** 1 Department of Psychology, University of Almería, 04120 Almería, Spain; msc943@ual.es (M.S.-C.); icuadrad@ual.es (I.C.-G.); arojas@ual.es (A.J.R.-T.); 2 Instituto de Neurociencias “F. Oloriz”, University of Granada, 18013 Granada, Spain; epita@ugr.es

**Keywords:** suicidal ideation, suicide, fibromyalgia, profiles, transdiagnostic psychological factors

## Abstract

Several studies have emphasized the heterogeneity of fibromyalgia patients. Furthermore, fibromyalgia patients are considered a high-risk suicide group. The ideation-to-action framework proposes a set of transdiagnostic psychological factors involved in the development of suicidal ideation. The present study aims to explore the existence of different subgroups according to their vulnerability to suicidal ideation through these transdiagnostic psychological variables and a set of variables typically associated with fibromyalgia. In this cross-sectional study, 151 fibromyalgia patients were assessed through the Revised Fibromyalgia Impact Questionnaire, Beck Depression Inventory-II, Plutchik Suicide Risk Scale, Interpersonal Needs Questionnaire, Defeat Scale, Entrapment Scale, Psychache Scale, and Beck Hopelessness Scale. A K-means cluster analysis identified two clusters, one (45.70%) according to a low vulnerability, and a second (54.30%) with a high vulnerability to suicidal ideation. These clusters showed statistically significant differences in suicidal ideation and suicide risk. However, no differences were observed in most socio-demographic variables. In conclusion, fibromyalgia patients who present a clinical condition characterized by a moderate-high degree of physical dysfunction, overall disease impact and intensity of fibromyalgia-associated symptoms, along with a high degree of perceived burdensomeness, thwarted belongingness, defeat, entrapment, psychological pain and hopelessness, form a homogeneous group at high risk for suicidal ideation.

## 1. Introduction

Fibromyalgia (FM) is a multisystemic syndrome characterized by the presence of generalized pain, tenderness, stiffness, and other associated symptoms such as fatigue, insomnia, anxiety, depression, and somatic complaints of various kinds [1,2]. The average global prevalence of FM is 2.4%, with notable variations by country; from 0.4% in Greece to 9.3% in Tunisia [3]. FM can affect any age and the female-to-male ratio is 8–10:1 [4], although most are women between 40 and 60 years old [5]. In addition, fibromyalgia syndrome is associated with a significant burden on the individual and society [6]. FM patients have a high co-morbidity with depression, anxiety, post-traumatic stress disorder, and obsessive-compulsive disorder [7]. Psychosocial and behavioral problems may contribute to and modify the presentation and treatment of fibromyalgia [7].

In general, the treatment of FM is symptomatic and requires a multidisciplinary approach combining non-pharmacological (e.g., physical exercise, psychotherapy, physical therapy, etc.) and pharmacological treatment [8,9]. Pharmacological treatment has not demonstrated a great improvement of all symptomatic domains, nor a large effect or higher efficacy than other treatments [6,10]. Half of FM patients are dissatisfied with the medication they receive [11] and only 25% of those who initiate pharmacological treatment continue it after one year [12]. Adherence to treatment is low and a significant proportion of patients do not receive adequate treatment [12]. Similarly, non-pharmacological treatment is perceived as ineffective and unsatisfactory [13,14]. There is no evidence of an increase in overall mortality among FM patients, nor a reduction in life expectancy [15], although an increase in suicides and accidental deaths compared to the general population is found [16].

There is evidence in the literature that groups with chronic pain, such as people with FM, are at high risk for suicide [17,18]. Additionally, the risk of suicide is higher in people with pain who have multiple physical and psychological symptoms [19]. Therefore, the study of the suicidal phenomenon in FM should be addressed through a comprehensive evaluation that includes the physical and psychological comorbidity of these patients.

In a systematic review of studies conducted primarily in the U.S. population, Tang and Crane [20] found that the lifetime prevalence of suicidal ideation in people with a clinical condition characterized by chronic pain (migraine, orthopedic patients with pain for more than seven months, rheumatoid arthritis, chronic abdominal pain, and chronic non-malignant pain) was approximately 20%, with notable differences between the different samples (from 5% of orthopedic patients to 50% of patients with chronic non-malignant pain). Likewise, some studies have shown a point prevalence of about 50% in FM patients [21].

Suicidal ideation is defined as the state characterized by thoughts or desires of being dead or ending one’s life [22]. Suicidal desire involves uneasiness and suffering for the affected person, often in silence [23]. Suicidal ideation may be a precedent to other more serious suicidal behaviors [23] and is the third strongest predictor of death by suicide [24]. Therefore, the study of factors involved in the development of suicidal ideation in FM patients is essential to reduce psychological distress and reduce other more severe suicidal behaviors.

Previous studies [25,26] have highlighted the need to go beyond psychiatric disorders to predict and explain suicidal ideation and other behaviors in patients with chronic pain [27]. A novel perspective on the conceptualization of the suicidal phenomenon is the ideation-to-action framework [28,29]. The main characteristic of this perspective is the understanding of the suicidal phenomenon in two differentiated processes: the development of suicidal ideation (and the variables essentially involved in this process) and the transition from ideation to suicidal action (and the variables primarily involved in this transition). The three predominant ideation-to-action models are: the interpersonal theory of suicide (IPTS; [30]), the integrated motivational-volitional model of suicide (IMV; [31]), and the three-step theory (3ST; [32]). All three models share this distinction (ideation vs. action) but differ in the key variables involved in each.

The first model that was developed within this theoretical perspective was the IPTS [30]. This model postulates that the development of suicidal ideation is contingent on the frustration of the need for relationship, which is operationalized in two variables: perceived burdensomeness (i.e., the belief of being a burden to oneself or others) and thwarted belongingness (i.e., the experience of feeling disconnected from valuable social circles, such as family or friends). The central hypothesis is that suicidal desire emerges when high levels of both variables are experienced [33]. In patients with chronic pain, perceived burdensomeness has been significantly associated with suicidal ideation [34,35]. In addition, Fishbain et al. [34] found a greater perceived burdensomeness in patients with chronic pain compared to patients with acute pain and a control group. Similarly, in a sample of FM patients, Lafuente-Castro et al. [36] showed that patients with suicidal ideation scored significantly higher in perceived burdensomeness than patients without suicidal ideation and the control group. Likewise, FM patients scored significantly higher in thwarted belongingness than the control group.

According to IMV [31], the key variables involved in the development of suicidal ideation/intention are defeat and entrapment. According to the cry of pain theory hypothesis [37], suicidal ideation/intention arises when people feel defeated (i.e., perceived failed struggle and helplessness as a result of a loss or significant alteration of social status, identity, or goals) and there is no perceived escape or rescue from such a situation (i.e., entrapment). While the main assumptions of IMV have been supported empirically in previous studies [38,39,40,41], they have not been tested in people with chronic pain [27].

Finally, the most recent ideation-to-action theory is 3ST [32]. According to this theory, pain and hopelessness are the most relevant variables involved in the development of suicidal ideation. The 3ST positions psychological pain (i.e., aversive and unbearable internal experience, composed of a combination of feelings such as shame, humiliation, loneliness, fear, or distress; [42]) as the type of pain most frequently involved in the emergence of suicidal ideation. Although experiencing chronic physical pain is clearly associated with increased suicidal risk, psychological pain has been shown to be a stronger predictor of suicide [26]. In addition, previous findings suggest that there is substantial overlap between the neural correlates of physical pain and psychological pain [43].

The multifaceted expression of FM, with marked variability in presentation and severity of symptoms, suggests that FM patients are a heterogeneous group [44,45]. Consequently, the variability of FM-associated symptoms has contributed to the more costly and time-consuming process of obtaining a diagnosis and, consequently, the difficulty of establishing appropriate treatment [46]. Some authors have found the existence of different FM subgroups based on physical and psychological/cognitive symptoms associated with fibromyalgia syndrome (see Perez-Aranda et al. [47], for a review). Studies such as that of Luciano et al. [48] identified three subgroups of FM patients: one subgroup with scores consistent with low-moderate severity on all physical and psychological variables (functional profile), a second subgroup with moderate-high scores on all variables and consistent with a dysfunctional profile, and a third subgroup similar to the dysfunctional profile but with higher scores on psychological variables such as depression, anxiety, and catastrophism. However, the same number of profiles is not always found. Studies by Vincent et al. [49] and Follick et al. [50] have identified four subgroups of FM patients: two extreme subgroups with high vs. low scores on all variables and two subgroups with moderate physical symptoms distinguishable from each other by degree of psychological condition (high vs. low). Likewise, studies based on more specific aspects, such as the psychopathological dimensions of personality, found the existence of two subgroups differentiated mainly by the degree (low vs. high) of negative emotionality/neuroticism, aggressiveness, restraint (i.e., the degree to which behavior is limited by consideration of future consequences) and introversion [51]. In this line, Torres et al. [52] found two subgroups of FM patients: one with a maladaptive personality profile (i.e., high predisposition to social distress, low positive affectivity, tendency to inhibitory behavior, less open to experience, low cooperative behavior, less proactive and with a tendency to maladaptive strategies for managing social conflicts) and another with scores in line with an adapted personality. Despite the differences in the number of subgroups and variables used, previous studies have found variability in the presence and intensity of symptoms associated with fibromyalgia syndrome. The heterogeneity presented by FM patients requires the establishment of profiles so that they can benefit from specific treatment (psychological and/or pharmacological) according to their characteristics. As we have seen, the prevalence and severity of suicidal ideation in this group is remarkable [19]. However, studies exploring the variables related to suicidal behavior in FM patients are scarce [53].

The present study aims to explore the existence of different subgroups of FM patients according to their vulnerability to suicidal ideation. To this end, key variables included in the ideation-to-action theories and that are essentially involved in the development of suicidal ideation (i.e., perceived burdensomeness, thwarted belongingness, defeat, entrapment, psychological pain, and hopelessness) will be taken into account, as well as variables essentially related to the fibromyalgia syndrome, such as physical function, overall impact of FM, intensity of physical pain and symptoms associated with FM and severity of depressive symptoms. This will allow us to establish profiles that cover the wide spectrum of physical and psychological symptoms of these patients, as well as to evaluate whether the degree of physical dysfunction or severity is consistent with the presence of high values in the variables involved in suicidal ideation and, therefore, could be part of a potential profile of patients vulnerable to suffering from it. The results of this work could orient psychological therapy towards new therapeutic targets that have not been explored among FM patients (e.g., perception of defeat and entrapment), as well as emphasize the relevance of a holistic approach (physical and psychological) of FM patients in the screening and treatment of suicidal ideation. According to the ideation-to-action framework, preventing suicidal ideation is reducing distress and preventing suicidal behavior, since the development of suicidal ideation is the first and most common step on the pathway to more serious suicidal behavior.

## 2. Materials and Methods

### 2.1. Participants

The selection of participants was carried out by incidental sampling. The sample consisted of a total of 151 FM patients residing in Spain (Mage = 49.82, SDage = 10.10, Range = 20–72 years old; 88.8% women) who completed an online questionnaire. The inclusion criteria were: (1) to be older than 18 years old, and (2) self-report being diagnosed with FM according to the criteria of the American College of Rheumatology 1990 [1]. No exclusion criteria were established. Sociodemographic characteristics can be seen in Table 1.

### 2.2. Instruments

Fibromyalgia Impact Questionnaire Revised (FIQ-R; [54]) was adapted to Spanish [55]. A self-administered questionnaire measures the overall impact of FM on patients’ quality of life. It consists of 21 items with visual analog scale (VAS) response options with a response range from 0 to 10. It is composed of three dimensions: (a) degree of difficulty of the physical activity (scores are obtained by adding the first nine items divided by 3, ranging from 0 to 30 points); (b) overall impact of FM (scores are obtained by adding items 10 and 11, ranging from 0 to 20 points); (c) intensity of FM-associated symptoms (scores are obtained by adding the last ten items divided by 2, ranging from 0 to 50 points). For this study, each of the dimensions was treated independently in order to assess their specific contribution (physical or psychological) to the classification of participants. In addition, the FM symptoms dimension does not include item 12 (pain), as it was treated as an indicator of physical pain intensity. In this paper, estimates of the reliability of the scores for each dimension using the Cronbach alpha coefficient were 0.94 (physical function), 0.88 (overall impact), and 0.87 (associated symptoms).

Beck Depression Inventory, version II (BDI-II; [56]) adapted to Spanish [57]. Self-administered 21-item scale with a multiple choice (between 0 and 3 points) to measure the severity of depressive symptoms. The BDI-II was used for this study by subtracting the score of item 9 (suicidal ideation), which was used as an indicator of suicidal ideation. The estimate of the reliability of the BDI-II scores was 0.90.

Plutchik Suicide Risk Scale (PSRS; [58]) adapted to Spanish [59]. Self-applied scale with 15 items that measures aspects related to suicidal behaviors. The response format is true/false. The internal consistency of the scale scores through Cronbach’s alpha coefficient was 0.75.

Interpersonal Needs Questionnaire (INQ-10; [60]) adapted to Spanish [61]. This self-administered 10-item questionnaire evaluates perceived burdensomeness (first six items) and thwarted belongingness (last four items). The response options are of the seven-point Likert type, ranging from 1 (not entirely true for me) to 7 (entirely true for me). The reliability estimate of the INQ-subscale scores through Cronbach’s alpha coefficient were 0.93 (perceived burdensomeness) and 0.84 (thwarted belongingness).

Defeat Scale (DS; [62]) adapted to Spanish [63]. Self-administered 16-item scale that measures how often participants have felt defeated. The response options are 5-point Likert type, from 0 (never) to 4 (always). In our sample, the reliability estimate of the defeat scale scores through Cronbach’s alpha coefficient was 0.95.

Entrapment scale (ES; [62]) adapted to Spanish [63]. This self-administered scale of 16 items evaluates the perception of entrapment. The response options are 5-point Likert type, from 0 (not quite like me) to 4 (extremely like me). The reliability estimate through Cronbach’s alpha coefficient was 0.96.

Psychache Scale (PS; [64]) adapted to Spanish [65]. Self-administered 13-item scale that assesses the degree of psychological pain. The response options to the first nine items range from 1 (never) to 5 (always). The response options to the last four items range from 1 (strongly disagree) to 5 (strongly agree). The estimate of the reliability of the scores through Cronbach’s alpha coefficient was 0.93.

Beck Hopelessness Scale (BHS; [66]) adapted to Spanish [67]. Self-administered 20-item scale that measures negative expectations about the future, well-being, and ability to cope with difficulties. The response format is true/false. The estimation of the reliability of the scores through Cronbach’s alpha coefficient was 0.91.

### 2.3. Procedure

The present study has a cross-sectional design. The managers of a group of FM patient associations located in different parts of Spain were contacted and they promoted and distributed among their members a questionnaire in line with the scales and socio-demographic questions of the study (http://encuestas.ual.es/limesurvey/index.php/539641?lang=es). Participation was anonymous and voluntary. All the participants gave their informed consent. They were provided with a contact address to answer any questions about their participation in the study. This study was approved by the Bioethics Committee on Human Research at University of Almería, Spain (Ref: UALBIO2018/018).

### 2.4. Data Analysis

A total of 185 participants began to fill out the questionnaire, which was completed in full by 147 participants. In addition, four participants completed all the scales, but not the socio-demographic data. Therefore, they were considered for analysis, making a total sample of 151 participants in this study. Eleven variables were used to form the subgroups. On the one hand, the physical and psychological variables specifically associated to the fibromyalgia syndrome: difficulty of physical activity (total score of the physical function dimension of the FIQ-R), overall impact of FM (total score of this dimension of the FIQ-R), intensity of FM associated symptoms (adjusted total score of this dimension of the FIQ-R, excluding item 12: physical pain), intensity of physical pain (score of item 12 of the FIQ-R; to specifically assess the contribution of the cardinal symptom of the fibromyalgia syndrome to cluster formation) and severity of depressive symptomatology (BDI-II adjusted without item 9: suicidal ideation). And, on the other hand, the variables involved essentially in the development of suicidal ideation of the IPTS (total score of each of the INQ subscales, perceived burdensomeness and thwarted belongingness), of the IMV (total score of the defeat scale and the entrapment scale) and of the 3ST (total score of the Psychache scale and the hopelessness scale). Prior to the cluster analysis, the scores of all variables were standardized.

**Figure 1:**
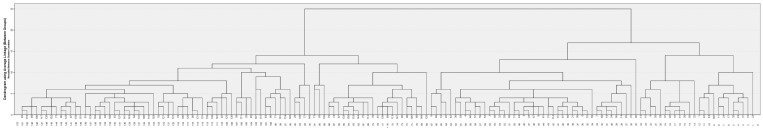
Dendrogram.

Due to the exploratory nature of the study, a hierarchical cluster analysis was first performed in which a dendrogram was obtained (Figure 1). This dendrogram is a graphic representation that summarizes the process of grouping the cases and provides guidance on how many clusters should be formed. Later, in order to obtain the profiles among FM patients, a K-means cluster analysis was performed, in which two clusters were specified according to the results shown by the dendrogram. The method selected was iteration and classification, a procedure that estimates the centroids iteratively and classifies the participants according to the estimated centroids. The iteration process was stopped when seven iterations were reached. Euclidean distance was used to measure the distance between cases. The result showed an ANOVA to check the relevance and separation of the variables between the clusters.

Also, as a cross validity procedure, three additional K-means cluster analyses were performed with a random case selection of 25%, 50%, and 75% of the total sample to compare the degree of stability between the different solutions. Likewise, to obtain validity evidence on the meaning of the profiles, we compared the suicidal ideation (item 9 of the BDI-II) and the suicidal risk (total score of the Plutchik Suicide Risk Scale) of both clusters through the U-Mann–Whitney test (suicidal ideation) and the Student *t*-test (suicidal risk). Finally, the independence of the sociodemographic characteristics of the participants was analyzed according to their belonging to each cluster through the Chi-squared test. All analyses were performed with IBM SPSS Statistics for Windows, Version 26.0. Armonk, NY, USA: IBM Corp.

## 3. Results

Since the dendrogram showed a clear solution of two clusters, a K-mean cluster analysis was executed requesting two clusters. The ANOVA (Table 2) shows that all variables contribute to form two distinguishable clusters (i.e., they show statistically significant differences). Likewise, the cross-validation process through the results obtained with 25%, 50% and 75% of the cases confirms the consistency of the final solution of two clusters.

**Figure 2:**
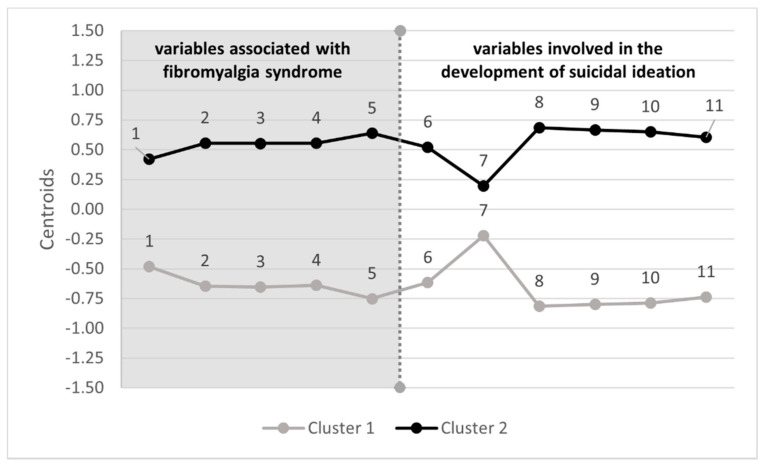
Profiles of suicidal ideation in patients with fibromyalgia. Note: On the left side of the dividing line are the variables specifically associated with fibromyalgia syndrome. On the right side are the variables essentially involved in the development of suicidal ideation; 1 = Physical function difficulty, 2 = Overall FM impact, 3 = Intensity of associated symptoms, 4 = Physical pain, 5 = Severity depressive symptomatology, 6 = Perceived burdensomeness, 7 = Thwarted belongingness, 8 = Defeat, 9 = Entrapment, 10 = Psychological pain, 11 = Hopelessness.

Cluster 1 (69 participants, 45.70%) is characterized by moderate values in the dimensions of FIQ-R (i.e., physical function difficulty, overall impact and intensity of associated symptoms) and in the item of physical pain intensity, mild-moderate values in the severity of depressive symptomatology, low values of perceived burdensomeness and thwarted belongingness, low values of perception of defeat and entrapment, moderate values of psychological pain and low hopelessness (Table 2). This finding is congruent with a profile of low vulnerability to suicidal ideation. Cluster 2 (82 participants, 54.30%) presents moderate-high values in the three dimensions of FIQ-R and high intensity of physical pain, values of severe depressive symptoms, moderate values of perception of perceived burdensomeness and thwarted belongingness, as well as high values in perception of defeat, entrapment, psychological pain and hopelessness (Table 2). It could be argued that cluster 2 adjusts to a profile of high vulnerability to suicidal ideation. See Figure 2 for a graphical interpretation of the profiles.

In order to obtain validity evidence on the meaning of the profiles, scores for suicidal ideation and risk were compared between both profiles. There are statistically significant differences (U = 4282.500; *p* < 0.001) in suicidal ideation between low profile (average range = 54.93) and high profile (average range = 93.73), with participants from the latter being the most vulnerable to suicidal ideation and therefore having higher values of suicidal ideation. In addition, using item 15 of Plutchik Suicide Risk Scale “Have you ever tried to take your own life?” in the low profile, 13% had tried to end their own life compared to 34.1% in the high profile who had done so (χ2 = 9.020; *p* = 0.003). Similarly, when comparing suicide risk, statistically significant differences are observed (t = −9.432; *p* < 0.001) between low profile (M = 6.01; SD = 2.62) and high profile (M = 9.93; SD = 2.47). Therefore, as with suicidal ideation, participants in high profile (high vulnerability to suicidal ideation) obtain higher values in suicidal risk.

Regarding the differences between profiles in the sociodemographic variables (Table 1), no statistically significant discrepancies are observed between the participants of both clusters in the frequencies of sex (χ2 = 3.870; *p* = 0.144), educational level completed (χ2 = 3.851; *p* = 0.278), marital status (χ2 = 4.967; *p* = 0.291) and religion (χ2 = 7.402; *p* = 0.192). However, work activity does show statistically significant dependence between both subgroups (χ2 = 16.922; *p* = 0.005), with a higher proportion of employees in low profile (43.5%) compared to high profile (32.1%), as well as a higher proportion of retirees/pensioners/rentiers in low profile than in high profile (26.1% vs. 9.0%). In contrast, high profile has higher frequencies of unemployed (25.6% vs. 13.0%) and permanent disability pensioners (15.4% vs. 2.9%) than low profile.

## 4. Discussion

The heterogeneous presence and severity of symptoms among FM patients limits understanding of fibromyalgia and makes it difficult to choose appropriate treatment [68]. Despite the high prevalence of suicidal ideation in these patients [21,53], as well as the numerous studies that have explored homogenous groups through the physical and psychological factors associated with the fibromyalgia syndrome [47], neither has sought to delineate subgroups of FM patients consistent with their vulnerability to suicidal ideation. Likewise, the study of suicide in FM has been based primarily on psychopathological comorbidity (e.g., depression), an approach that has proven unsuccessful in predicting suicide and has deprived it of its own entity [24].

The present study aimed to explore the existence of homogeneous subgroups of FM patients according to their vulnerability to suicidal ideation. The main novelty is the use of psychological variables of transdiagnostic relevance (i.e., involved in a high range of psychological disorders) and considered as key elements in the development of suicidal ideation of the three predominant theories (i.e., IPTS, IMV, and 3ST) of the ideation-to action-framework. A cluster analysis was carried out, consisting of these psychological variables (i.e., perceived burdensomeness, thwarted belongingness, defeat, entrapment, psychological pain, and hopelessness) together with other variables associated with fibromyalgia syndrome (i.e., difficulty of physical function, overall impact of FM, intensity of associated symptoms, intensity of physical pain, and severity of depressive symptoms). Our findings show that, as expected, these variables adequately identify and discriminate fibromyalgia patients who may be at risk of suffering from suicidal ideation, reducing the variability to two groups (high risk/low risk), despite the heterogeneity of these patients. Additionally, the variables of the theories behave in a similar and concomitant way, showing a high relationship among them and their association with the intensity of the typical symptoms of fibromyalgia (e.g., pain), physical functioning, and impact. In short, this is an exploratory study that positions a set of variables to be examined among fibromyalgia patients and a claim for exploring the theoretical assumptions of these theories to clarify the high prevalence of suicidal ideation in fibromyalgia and to optimize screening and psychological management.

The result showed a solution of two clusters (or profiles) of FM patients. A first profile characterized by patients with low-moderate values in all the variables implemented in the analysis, affected to a lesser extent by the fibromyalgia syndrome, physically and psychologically, and with low values in the psychological variables involved in suicidal ideation. Therefore, this is a group of participants that is predictably less vulnerable to suffering suicidal ideation. It should be noted that this subgroup represented 45% of the sample, so that more than half of the FM patients (cluster 2) obtained scores reflecting more severe physical and psychological symptoms and scores on the variables related to suicidal ideation comparable to a high risk of suffering from suicidal ideation. Although this proportion does not show the specific prevalence of suicidal ideation in these patients (48% in the study by Calandre et al. [21], or 32.5% in the study by Triñanes et al. [53]), it denotes a marked vulnerability that makes screening and follow-up of suicidal ideation and other behaviors in FM patients in the clinical setting indispensable.

In relation to the profile of high vulnerability to suicidal ideation, according to Calati et al. [19], patients with chronic pain who are at high suicidal risk tend to present a highly comorbid and more exacerbated clinical condition (physical and psychological), so a subgroup with higher values in the variables associated with fibromyalgia syndrome and in line with higher values in the variables involved in suicidal ideation was expected. This finding is consistent with the result of Triñanes et al. [53] about the existence of a positive and statistically significant association between the impact of FM on daily activities and the presence of suicidal ideation. Similarly, Calandre et al. [69] found a positive and statistically significant association between the FIQ score and suicidal risk.

Regarding the variables involved in suicidal ideation, previous studies have shown that people with chronic pain are perceived to be more of a burden compared to people with acute pain and the control groups [34]. Similarly, Wilson et al. [35] found a positive and statistically significant association between perceived burdensomeness and suicidal ideation. With a sample of FM patients, a study by Lafuente-Castro et al. [36] showed a higher score for perceived burdensomeness and thwarted belongingness in FM and suicidal ideation patients compared to non-suicidal ideation patients and controls. However, in our work, despite statistically significant differences in thwarted belongingness between the two profiles, the size of this difference is the smallest. This result is in line with the growing empirical evidence that perceived burdensomeness is more related to suicidal ideation than thwarted belongingness [70,71].

To our knowledge, the perception of defeat and entrapment has not yet been explored in chronic pain patients. However, there are studies [72,73] that have evaluated a construct similar to defeat in a long-term, intensely painful population: mental defeat (i.e., a psychological state characterized by a loss of autonomy, individual agency, and human integrity; [74]). Tang et al. [74] found higher levels of mental defeat in patients with chronic pain compared to those with acute pain and those without pain. A positive, moderate, and statistically significant association has also been found between mental defeat and the worst suicide attempt [73]. The conceptual and methodological overlap between perception of defeat and mental defeat [27], together with the high perception of defeat presented by FM patients in the profile of high vulnerability to suicidal ideation, suggests a similar functioning of both constructs in people with chronic pain. Given that IMV [31] is one of the most prominent models in the field of suicide, the study of key variables and their association with the characteristics of the chronic pain population may help to explain suicidal ideation in these patients. A meta-analysis by Ducasse et al. [75] found that the relationships between psychological pain and ideation and other suicidal behaviors are significant even in controlling depression. Similarly, patients with suicidal ideation also scored higher in psychological pain compared to patients without suicidal ideation. A systematic review by Verrocchio et al. [76] states that psychological pain may be the most significant predictor of suicidal ideation. However, to our knowledge, there is no research that has evaluated this variable in FM patients. In our study, participants in the high vulnerability to suicidal ideation cluster had high scores for psychological pain, which may be further evidence of the severity of their suicidal ideation, or their potential vulnerability to it. These patients also show high scores for hopelessness. Interestingly, despite studies that position hopelessness as a strong predictor of suicidal ideation [69], the scientific literature on fibromyalgia syndrome has not delved into the role of this variable in suicidal ideation and behavior in FM patients.

Finally, we found no statistically significant differences in the sociodemographic variables between patients in both subgroups, except for work activity. Our findings are consistent with those of Smith et al. [77]: sociodemographic variables are less associated with ideation and other suicidal behaviors in patients with chronic pain. However, FM patients were 1.9 times more likely to be absent from work due to their illness than their colleagues [78]. In the present study, the profile of high vulnerability to suicidal ideation consisted of a higher proportion of FM patients who were unemployed and permanently disabled. Since these patients are the most affected by their physical and psychological symptoms, it is understandable that they are not in a suitable condition to work. Similarly, unemployed or permanently disabled status may be a stressor that influences the impact of the disease [53].

This study is not exempt from limitations. Suicidal ideation was evaluated with only one indicator (item 9 of the BDI-II). However, despite the limitations related to the capability of a single item to capture the complexity of the construct, this item has been used frequently in the literature and it has an outstanding reputation based on the concurrent validity with the Beck Scale for Suicidal Ideation [79]. Another limitation is that the sample of FM patients was recruited from associations of fibromyalgia patients located throughout Spain, which could lead to coverage bias. Therefore, we urge caution in generalizing our findings to all FM patients. Finally, the cross-sectional design of the research makes it impossible to detect possible temporal fluctuations and the influence of context, which could have an impact on all the variables analyzed. Longitudinal studies would provide information regarding this temporal fluctuation and its possible impact on the development of suicidal ideation in FM patients. Similarly, future studies should evaluate the contribution of other variables (e.g., attachment styles) that have been shown to be related to ideation and suicidal behavior in high-risk suicidal samples and fibromyalgia patients [80,81,82,83], as well as explore the relationship of these variables and the key elements of ideation-to-action theories.

## 5. Conclusions

In conclusion, FM patients who present a clinical condition characterized by a moderate to high degree of physical dysfunction, overall disease impact and intensity of FM-associated symptoms (including high severity of depressive symptoms), along with a high degree of perceived burdensomeness, thwarted belongingness, defeat, entrapment, psychological pain, and hopelessness, form a homogeneous group at high risk for suicidal ideation. According to the ideation-to-action framework, preventing suicidal ideation is not only about preventing the suffering and distress that it causes, but also limiting the transition to suicidal action [23,84]. Therefore, in the clinical setting, assessing the variables involved in the development of suicidal ideation could improve the screening and management of patients with fibromyalgia. Being able to discern between those patients who are at risk of suffering from suicidal ideation and to orient psychological intervention to the concrete variables, as well as to evaluate the effectiveness of psychological therapy with pre-post measures. Likewise, we believe it is relevant that in the out-of-hospital context (e.g., associations of patients with fibromyalgia), the variables involved in suicidal ideation in patients with fibromyalgia should be evaluated in order to interfere in the development as soon as possible, since only a third of the people who die by suicide had attended mental health services in the year prior to the suicide [85]. Similarly, in the research field, ideation-to-action theories (i.e., IPTS, IMV, and 3ST) reconceptualize the suicidal phenomenon and propose a set of transdiagnostic psychological variables that could clarify the relationship between chronic pain and suicidal ideation and other suicidal behaviors.

## Figures and Tables

**Table 1:** Socio-demographic characteristics of the participants.

	Total [*N* (%)]	Cluster 1 (Low Vulnerability) [*N* (%)]	Cluster 2 (High Vulnerability) [*N* (%)]	*p*-Value (Cluster 1 vs. Cluster 2)
Age	*M* = 49.82; SD = 10.10	*M* = 50.25; *SD* = 11.15	*M* = 49.45; *SD* = 9.13	0.634 *
Sex				0.144 **
Woman	134 (91.2%)	64 (92.8%)	70 (89.7%)	
Man	13 (8.8%)	5 (7.2%)	8 (10.3%)	
Completed education level				0.278 **
No studies	3 (2%)	1 (1.4%)	2 (2.6%)	
Primary education	20 (13.6%)	8 (11.6%)	12 (15.4%)	
Secondary education	80 (54.4%)	34 (49.3%)	46 (59.0%)	
Higher education	44 (29.9%)	26 (37.7%)	18 (23.1%)	
Work activity				0.005 **
Unemployed	29 (19.7%)	9 (13.0%)	20 (25.6%)	
Student	4 (2.7%)	2 (2.9%)	2 (2.6%)	
Homemaker	20 (13.6%)	8 (11.6%)	12 (15.4%)	
Employee	55 (37.4%)	30 (43.5%)	25 (32.1%)	
Sick leaved	14 (9.5%)	2 (2.9%)	12 (15.4%)	
Retired/Pensioner/Rentist	25 (17.0%)	18 (26.1%)	7 (9.0%)	
Civil status				0.291 **
Single	28 (20.1%)	13 (19.4%)	15 (20.8%)	
Stable partner	15 (10.8%)	6 (9.0%)	9 (12.5%)	
Married	76 (54.7%)	42 (62.7%)	34 (47.2%)	
Divorced	18 (12.9%)	5 (7.5%)	13 (18.1%)	
Widowed	2 (1.4%)	1 (1.5%)	1 (1.4%)	
Religion				0.192 **
Catholic	76 (53.9%)	36 (52.9%)	40 (54.8%)	
Orthodox	2 (1.4%)	2 (2.9%)	-	
Protestant	2 (1.4%)	2 (2.9%)	-	
Agnostic	13 (9.2%)	8 (11.8%)	5 (6.8%)	
Atheist	24 (17.0%)	8 (11.8%)	16 (21.9%)	
Indifferent	24 (17.0%)	12 (17.6%)	12 (16.4%)	

Note: SD = standard deviation; * Student’s *t*-test, ** Chi-squared Test.

**Table 2:** Mean and standard deviation, centroids, and ANOVA for the solution of two clusters.

Variables	Cluster 1 (Low Vulnerability)		Cluster 2 (High Vulnerability)		F	*p*-Value
	Centroids	Mean (Standard Deviation)	Centroids	Mean (Standard Deviation)		
Physical function difficulty (FIQ-R)	−0.48	15.38 (7.08)	0.42	21.69 (5.39)	38.526	<0.001
Overall FM impact (FIQ-R)	−0.64	9.54 (4.88)	0.56	15.80 (3.48)	84.251	<0.001
Intensity of associated symptoms (FIQ-R)	−0.65	22.30 (8.75)	0.55	33.54 (6.24)	84.291	<0.001
Physical pain (FIQ-R)	−0.64	5.71 (2.38)	0.56	8.60 (1.46)	83.429	<0.001
Severity depressive symptomatology (BDI-II)	−0.75	19.74 (6.91)	0.64	34.89 (8.67)	137.259	<0.001
Perceived burdensomeness (INQ)	−0.62	10.52 (4.81)	0.52	22.33 (10.79)	70.676	<0.001
Thwarted belongingness (INQ)	−0.22	13.80 (6.53)	0.19	16.49 (6.19)	6.730	0.010
Defeat (DS)	−0.82	21.13 (10.12)	0.69	42.77 (9.17)	189.687	<0.001
Entrapment (ES)	−0.80	18.87 (11.07)	0.67	42.46 (11.00)	171.460	<0.001
Psychological Pain (PS)	−0.79	32.94 (9.12)	0.65	49.80 (7.27)	159.770	<0.001
Hopelessness (BHS)	−0.74	5.59 (4.32)	0.61	13.55 (4.46)	122.793	<0.001

Note: FIQ-R = Fibromyalgia Impact Questionnaire Revised; BDI-II = Beck Depression Inventory, version II; INQ = Interpersonal Needs Questionnaire; DS = Defeat scale; ES = Entrapment scale; PS = Psychache scale; BHS = Beck Hopelessness scale.

## Data Availability

The data presented in this study are available on request from the corresponding author. The data are not publicly available due to privacy.

## References

[1] Wolfe F., Smythe H.A., Yunus M.B., Bennett R.M., Bombardier C., Goldenberg D.L., Tugwell P., Campbell S.M., Abeles M., Clark P. (1990). The American college of rheumatology 1990 criteria for the classification of fibromyalgia. Arthritis Rheum..

[2] Wolfe F., Clauw D.J., Fitzcharles M.A., Goldenberg D.L., Katz R.S., Mease P., Russell A.S., Russell I.J., Winfield J.B., Yunus M.B. (2010). The American College of Rheumatology Preliminary Diagnostic Criteria for Fibromyalgia and Measurement of Symptom Severity. Arthritis Care Res..

[3] Queiroz L.P. (2013). Worldwide Epidemiology of Fibromyalgia. Curr. Pain Headache Rep..

[4] Häuser W., Ablin J., Fitzcharles M.A., LittleJohn G., Luciano J.V., Usui C., Walitt B. (2015). Fibromyalgia. Nat. Rev. Dis. Primers.

[5] Häuser W., Fitzcharles M.A. (2018). Facts and myths pertaining to fibromyalgia. Dialogues Clin. Neurosci..

[6] Rico-Villademoros F., Postigo-Martin P., Garcia-Leiva J.M., Ordoñez-Carrasco J.L., Calandre E.P. (2020). Patterns of pharmacologic and non-pharmacologic treatment, treatment satisfaction and perceived tolerability in patients with fibromyalgia: A patients’ survey. Clin. Exp. Rheumatol..

[7] Cohen H. (2017). Controversies and challenges in fibromyalgia: A review and a proposal. Ther. Adv. Musculoskelet. Dis..

[8] Fitzcharles M.A., Ste-Marie P.A., Goldenberg D.L., Pereira J.X., Abbey S., Choinière M., Ko G., Moulin D.E., Panopalis P., Proulx J. (2013). 2012 Canadian Guidelines for the diagnosis and management of fibromyalgia syndrome: Executive summary. Pain Res. Manag..

[9] MacFarlane G.J., Kronisch C., Dean L.E., Atzeni F., Häuser W., Flub E., Choy E., Kosek E., Amris K., Branco J. (2017). EULAR revised recommendations for the management of fibromyalgia. Ann. Rheum. Dis..

[10] Calandre E.P., Rico-Villademoros F., Slim M. (2017). Pharmacological treatment of fibromyalgia: Is the glass half empty or half full?. Pain Manag..

[11] McNett M., Goldenberg D., Schaefer C., Hufstader M., Baik R., Chandran A., Zlateva G. (2011). Treatment patterns among physician specialties in the management of fibromyalgia: Results of a cross-sectional study in the United States. Curr. Med. Res. Opin..

[12] Liu Y., Qian C., Yang M. (2016). Treatment Patterns Associated with ACR-Recommended Medications in the Management of Fibromyalgia in the United States. J. Manag. Care Spec. Pharm..

[13] Bennett R.M., Jones J., Turk D.C., Russell I.J., Matallana L. (2007). An internet survey of 2596 people with fibromyalgia. BMC Musculoskelet. Disord..

[14] Taylor S.J., Steer M., Ashe S.C., Furness P.J., Haywood-Small S., Lawson K. (2019). Patients’ perspective of the effectiveness and acceptability of pharmacological and non-pharmacological treatments of fibromyalgia. Scand. J. Pain.

[15] Petzke F., Brückle W., Eidmann U., Heldmann P., Köllner V., Kühn T., Kühn-Becker H., Strunk-Richter M., Schiltenwolf M. (2017). General treatment principles, coordination of care and patient education in fibromyalgia syndrome: Updated guidelines 2017 and overview of systematic review articles. Schmerz.

[16] Wolfe F., Hassett A.L., Walitt B., Michaud K. (2011). Mortality in fibromyalgia: A study of 8186 patients over thirty-five years. Arthritis. Care Res..

[17] Campbell G., Darke S., Bruno R., Degenhardt L. (2015). The prevalence and correlates of chronic pain and suicidality in a nationally representative sample. Aust. N. Z. J. Psychiatry.

[18] Stenager E., Christiansen E., Handberg G., Jensen B. (2014). Suicide attempts in chronic pain patients. A register-based study. Scand. J. Pain.

[19] Calati R., Laglaoui-Bakhiyi C., Artero S., Ilgen M., Courtet P. (2015). The impact of physical pain on suicidal thoughts and behaviors: Meta-analyses. J. Psychiatr. Res..

[20] Tang N.K.Y., Crane C. (2006). Suicidality in chronic pain: A review of the prevalence, risk factors and psychological links. Psychol. Med..

[21] Calandre E.P., Navajas-Rojas M.A., Ballesteros J., García-Carrillo J., García-Leiva J., Rico-Villademoros F. (2014). Suicidal Ideation in Patients with Fibromyalgia: A Cross-Sectional Study. Pain Pract..

[22] Posner K., Oquendo M.A., Gould M., Stanley B., Davies M. (2007). Columbia Classification Algorithm of Suicide Assessment (C-CASA): Classification of Suicidal Events in the FDA’s Pediatric Suicidal Risk Analysis of Antidepressants. Am. J. Psychiatry.

[23] Jobes D.A., Joiner T.E. (2019). Reflections on Suicidal Ideation. Crisis.

[24] Franklin J.C., Ribeiro J.D., Fox K.R., Bentley K.H., Kleiman E.M., Huang X., Musacchio K.M., Jaroszewski A.C., Chang B.P., Nock M.K. (2017). Risk factors for suicidal thoughts and behaviors: A meta-analysis of 50 years of research. Psychol. Bull..

[25] Hassett A.L., Aquino J.K., Ilgen M.A. (2014). The Risk of Suicide Mortality in Chronic Pain Patients. Curr. Pain Headache Rep..

[26] Hooley J.M., Franklin J.C., Nock M.K. (2014). Chronic Pain and Suicide: Understanding the Association. Curr. Pain Headache Rep..

[27] Kirtley O.J., Rodham K., Crane C. (2020). Understanding suicidal ideation and behaviour in individuals with chronic pain: A review of the role of novel transdiagnostic psychological factors. Lancet Psychiatry.

[28] Klonsky E.D., Saffer B.Y., Bryan C.J. (2018). Ideation-to-action theories of suicide: A conceptual and empirical update. Curr. Opin. Psychol..

[29] O’Connor R.C., Portzky G. (2018). Looking to the Future: A Synthesis of New Developments and Challenges in Suicide Research and Prevention. Front. Psychol..

[30] Joiner T.E. (2005). Why People Die by Suicide.

[31] O’Connor R.C. (2011). The Integrated Motivational-Volitional Model of Suicidal Behavior. Crisis.

[32] Klonsky E.D., May A.M. (2015). The Three-Step Theory (3ST): A New Theory of Suicide Rooted in the “Ideation-to-Action” Framework. Int. J. Cogn. Ther..

[33] Van Orden K.A., Witte T.K., Cukrowicz K.C., Braithwaite S.R., Selby E.A., Joiner T.E. (2010). The interpersonal theory of suicide. Psychol. Rev..

[34] Fishbain D.A., Bruns D., Bruns A., Gao J., Lewis J.E., Meyer L.J., Disorbio J.M. (2016). The Perception of Being a Burden in Acute and Chronic Pain Patients Is Associated with Affirmation of Different Types of Suicidality. Pain Med..

[35] Wilson K.G., Heenan A., Kowal J., Henderson P., McWilliams L.A., Castillo D. (2017). Testing the Interpersonal Theory of Suicide in Chronic Pain. Clin. J. Pain.

[36] Lafuente-Castro C.P., Ordóñez-Carrasco J.L., García-Leiva J.M., Salgueiro-Macho M., Calandre E.P. (2018). Perceived burdensomeness, thwarted belongingness and suicidal ideation in patients with fibromyalgia and healthy subjects: A cross-sectional study. Rheumatol. Int..

[37] Williams J.M.G., Pollock L.R., van Heeringen K. (2001). Psychological Aspects of the Suicidal Process. Understanding Suicidal Behaviour: The Suicidal Process Approach to Research, Treatment and Prevention.

[38] Dhingra K., Boduszek D., O’Connor R.C. (2015). Differentiating suicide attempters from suicide ideators using the Integrated Motivational–Volitional model of suicidal behaviour. J. Affect. Disord..

[39] Dhingra K., Boduszek D., O’Connor R.C. (2016). A structural test of the Integrated Motivational-Volitional model of suicidal behaviour. Psychiatry Res..

[40] Tucker R.P., O’Connor R.C., Wingate L.R. (2016). An Investigation of the Relationship between Rumination Styles, Hope, and Suicide Ideation Through the Lens of the Integrated Motivational-Volitional Model of Suicidal Behavior. Arch. Suicide Res..

[41] Wetherall K., Robb K.A., O’Connor R.C. (2018). An Examination of Social Comparison and Suicide Ideation through the Lens of the Integrated Motivational-Volitional Model of Suicidal Behavior. Suicide Life-Threat Behav..

[42] Shneidman E.S. (1993). Suicide as Psychache: A Clinical Approach to Self-Destructive Behavior.

[43] Elman I., Borsook D., Volkow N.D. (2013). Pain and suicidality: Insights from reward and addiction neuroscience. Prog. Neurobiol..

[44] Giesecke T., Williams D.A., Harris R.E., Cupps T.R., Tian X., Tian T.X., Gracely R.H., Clauw D.J. (2003). Subgrouping of fibromyalgia patients on the basis of pressure-pain thresholds and psychological factors. Arthritis Rheum..

[45] Turk D.C., Okifuji A., Sinclair J.D., Starz T.W. (1998). Differential responses by psychosocial subgroups of fibromyalgia syndrome patients to an interdisciplinary treatment. Arthritis Care Res..

[46] Wilson H.D., Robinson J.P., Turk D.C. (2009). Toward the identification of symptom patterns in people with fibromyalgia. Arthritis Rheum..

[47] Pérez-Aranda A., Andrés-Rodríguez L., Feliu-Soler A., Núñez C., Stephan-Otto C., Pastor-Mira M.A., López-Roig S., Peñacoba C., Calandre E.P., Slim M. (2019). Clustering a large Spanish sample of patients with fibromyalgia using the Fibromyalgia Impact Questionnaire–Revised. Pain.

[48] Luciano J.V., Forero C.G., Cerdà-Lafont M., Peñarrubia-María T., Fernández-Vergel R., Cuesta-Vargas A.I., Ruíz J.M., Rozadilla-Sacanell A., Sirvent-Alierta E., Santo-Panero P. (2016). Functional Status, Quality of Life, and Costs Associated with Fibromyalgia Subgroups. Clin. J. Pain.

[49] Vincent A., Hoskin T.L., Whipple M.O., Clauw D.J., Barton D.L., Benzo R.P., Williams D.A. (2014). OMERACT-based fibromyalgia symptom subgroups: An exploratory cluster analysis. Arthritis Res. Ther..

[50] Follick B.T., Cherry B.J., Rutledge D.N., Zettel-Watson L., Jones J.C. (2016). Heterogeneity in fibromyalgia based upon cognitive and physical performance and psychological symptomology. J. Am. Assoc. Nurse Pract..

[51] Gonzalez B., Novo R., Ferreira A.S. (2019). Fibromyalgia: Heterogeneity in personality and psychopathology and its implications. Psychol. Health Med..

[52] Torres X., Bailles E., Valdes M., Gutierrez F., Peri J.M., Arias A., Gomez E., Collado A. (2013). Personality does not distinguish people with fibromyalgia but identifies subgroups of patients. Gen. Hosp. Psychiatry.

[53] Triñanes Y., González-Villar A., Gómez-Perretta C., Carrillo-de-la-Peña M.T. (2014). Suicidality in Chronic Pain: Predictors of Suicidal Ideation in Fibromyalgia. Pain Pract..

[54] Bennett R.M., Friend R., Jones K.D., Ward R., Han B.K., Ross R.L. (2009). The Revised Fibromyalgia Impact Questionnaire (FIQR): Validation and psychometric properties. Arthritis Res. Ther..

[55] Salgueiro M., García-Leiva J.M., Ballesteros J., Hidalgo J., Molina R., Calandre E.P. (2013). Validation of a Spanish version of the Revised Fibromyalgia Impact Questionnaire (FIQR). Health Qual. Life Outcomes.

[56] Beck A.T., Steer R., Brown G. (1996). Manual for the Beck Depression Inventory-II.

[57] Sanz J., García-Vera M.P. (2013). Rendimiento diagnóstico y estructura factorial del Inventario para la Depresión de Beck–II (BDI-II). An. Psicol..

[58] Plutchik R., van Praag H.M., Conte H.R., Picard S. (1989). Correlates of suicide and violence risk 1: The suicide risk measure. Compr. Psychiat..

[59] Rubio G., Montero I., Jáuregui J., Villanueva R., Marin J.J., Santodomingo J. (1998). Validación de la escala de riesgo suicida de Plutchik en población española. Arch. Neurobiol..

[60] Van Orden K., Cukrowicz K., Witte T., Joiner T.E. (2012). Thwarted belongingness and perceived burdensomeness: Construct validity and psychometric properties of the Interpersonal Needs Questionnaire. Psychol. Assess..

[61] Ordóñez-Carrasco J.L., Salgueiro M., Sayans-Jiménez P., Blanc-Molina A., García-Leiva J.M., Calandre E.P., Rojas A.J. (2018). Psychometric properties of the Spanish version of the 12-item Interpersonal Needs Questionnaire in fibromyalgia syndrome patients. An. Psicol..

[62] Gilbert P., Allan S. (1998). The role of defeat and entrapment (arrested flight) in depression: An exploration of an evolutionary view. Psychol. Med..

[63] Ordóñez-Carrasco J.L., Cuadrado I., Rojas A.J. Adaptación al español de las escalas de derrota y atrapamiento en jóvenes adultos: Propiedades psicométricas. Ter. Psicol..

[64] Holden R.R., Mehta K., Cunningham E.J., McLeod L.D. (2001). Development and preliminary validation of a scale of psychache. Can. J. Behav. Sci..

[65] Ordóñez-Carrasco J.L., Cuadrado I., Rojas A.J. (2019). Escala de dolor psicológico: Adaptación de la Psychache Scale al español en jóvenes adultos. Rev. Psiquiatr. Salud Ment..

[66] Beck A.T., Weissman A., Lester D., Trexler L. (1974). The measurement of pessimism: The hopelessness scales. J. Consult. Clin. Psychol..

[67] Aguilar E.J., Hidalgo M.D., Cano R., López J., Campillo M., Hernández J. (1995). Estudio prospectivo de la desesperanza en pacientes psicóticos: Características psicométricas de la Escala de Desesperanza de Beck. An. Psiquiatr..

[68] Salaffi F., Mozzani F., Draghessi A., Atzeni F., Catellani R., Ciapetti A., Di Carlo M., Sarzi-Puttini P. (2016). Identifying the symptom and functional domains in patients with fibromyalgia: Results of a cross-sectional Internet-based survey in Italy. J. Pain Res..

[69] Calandre E.P., Vilchez J.S., Molina-Barea R., Tovar M.I., García-Leiva J.M., Hidalgo J., Rodríguez-López C.M., Rico-Villademoros F. (2011). Suicide attempts and risk of suicide in patients with fibromyalgia: A survey in Spanish patients. Rheumatology.

[70] Chu C., Buchman-Schmitt J.M., Stanley I.H., Hom M.A., Tucker R.P., Hagan C.R., Rogers M.L., Podlogar M.C., Chiurliza B., Ringer F.B. (2017). The interpersonal theory of suicide: A systematic review and meta-analysis of a decade of cross-national research. Psychol. Bull..

[71] Ma J., Batterham P.J., Calear A.L., Han J. (2016). A systematic review of the predictions of the Interpersonal–Psychological Theory of Suicidal Behavior. Clin. Psychol. Rev..

[72] Hazeldine-Baker C.E., Salkovskis P.M., Osborn M., Gauntlett-Gilbert J. (2018). Understanding the link between feelings of mental defeat, self-efficacy and the experience of chronic pain. Br. J. Pain.

[73] Tang N.K.Y., Beckwith P., Ashworth P. (2016). Mental Defeat Is Associated with Suicide Intent in Patients with Chronic Pain. Clin. J. Pain.

[74] Tang N.K.Y., Salkovskis P.M., Hanna M. (2007). Mental Defeat in Chronic Pain: Initial Exploration of the Concept. Clin. J. Pain.

[75] Ducasse D., Holden R.R., Boyer L., Artéro S., Calati R., Guillaume S., Courtet P., Olié E. (2018). Psychological Pain in Suicidality: A Meta-Analysis. J. Clin. Psychiatry.

[76] Verrocchio M.C., Carrozzino D., Marchetti D., Andreasson K., Fulcheri M., Bech P. (2016). Mental Pain and Suicide: A Systematic Review of the Literature. Front. Psychiatry.

[77] Smith M.T., Edwards R.R., Robinson R.C., Dworkin R.H. (2004). Suicidal ideation, plans, and attempts in chronic pain patients: Factors associated with increased risk. Pain.

[78] Kivimaki M., Leino-Arjas P., Kaila-Kangas L., Virtanen M., Elovainio M., Puttonen S., Keltikangas-Järvinen L., Pentti J., Vahtera J. (2006). Increased absence due to sickness among employees with fibromyalgia. Ann. Rheum. Dis..

[79] Beck A.T., Brown G.K., Steer R.A. (1997). Psychometric characteristics of the Scale for Suicide Ideation with psychiatric outpatients. Behav. Res. Ther..

[80] Green J., Berry K., Danquah A., Pratt D. (2020). The role of psychological and social factors in the relationship between attachment and suicide: A systematic review. Clin. Psychol. Psychother..

[81] Li S., Galynker I.I., Briggs J., Duffy M., Frechette-Hagan A., Kim H.J., Cohen L.J., Yaseen Z.S. (2017). Attachment style and suicide behaviors in high risk psychiatric inpatients following hospital discharge: The mediating role of entrapment. Psychiatry Res..

[82] Romeo A., Di Tella M., Ghiggia A., Tesio V., Fusaro E., Geminiani G.C., Castelli L. (2020). Attachment style and parental bonding: Relationships with fibromyalgia and alexithymia. PLoS ONE.

[83] Sechi C., Vismara L., Brennstuhl M.J., Tarquinio C., Lucarelli L. (2020). Adult attachment styles, self-esteem, and quality of life in women with fibromyalgia. Health Psychol. Open.

[84] Kleiman E.M. (2020). Suicidal thinking as a valuable clinical endpoint. EClinicalMedicine.

[85] Luoma J.B., Martin C.E., Pearson J.L. (2002). Contact with mental health and primary care providers before suicide: A review of the evidence. Am. J. Psychiatry.

